# Contribution of human oocyte architecture to success of in vitro maturation technology

**Published:** 2013-01

**Authors:** Mohammad Ali Khalili, Stefania A Nottola, Abbas Shahedi, Guido Macchiarelli

**Affiliations:** 1 *Research and Clinical Center for Infertility, Shahid Sadoughi University of Medical Sciences, Yazd, Iran.*; 2 *Department of Anatomy, Histology, Forensic Medicine and Orthopaedics, La Sapienza University, Rome, Italy. *; 3 *Department of Anatomy, Yazd Branch, Islamic Azad University, Yazd, Iran.*; 4 *Department of Life, Health and Environmental Sciences, University of L’Aquila, L’Aquila, Italy.*

**Keywords:** *Human*, *Oocyte*, *In Vitro Oocyte Maturation Technique*, *Cell Morphology*, *Ultrastructure*

## Abstract

The use of ovarian stimulation for infertility treatment is associated with side effects of ovarian hyperstimulation syndrome (OHSS) and potential cancer risk. This is also true in high risk women such as those polycystic with ovary (PCO) and polycystic ovarian syndrome (PCOS). In vitro maturation (IVM) of oocytes was primarily developed to make IVF safe for women with PCO and at high risk of OHSS. The application of IVM of oocytes to assist clinical infertility treatment remains poor because of the reduced developmental competence of oocytes after IVM, despite several decades of research. Reduced meiotic maturation and fertilization rates, as well as low blastocyst production reveal short-term developmental insufficiency of oocytes when compared with in vivo-matured counterparts. In this review, the structural role of human oocytes, revealed by different technical approaches, to the success of IVM technology is highlighted.

## Introduction

**Clinical aspects of In vitro maturation**

Since the birth of the first *In Vitro *Fertilization (IVF) baby, there have been improvements in the pregnancy and birth rates with IVF. Improvements in the birth rates have been attributed to the advances in hormonal stimulation of patients with various controlled ovarian hyperstimulation (COH) protocols and improved in vitro culture systems for gametes and embryos (1). 

However, the use of ovarian stimulation increases the patients’ cost and suffering, and is associated with side effects including ovarian hyperstimulation syndrome (OHSS) and potential cancer risk. This is especially true in high risk women such as those polycystic with ovary (PCO) and polycystic ovarian syndrome (PCOS) (2). The recovery of immature oocytes followed by in vitro maturation (IVM) and fertilization is an attractive alternative to IVF. Clinical IVM has several advantages of reducing the costs, avoidance of the side effects of OHSS and simplified treatment for certain infertile couples (3). Application of IVM is growing in companion animal breeding, livestock genetic improvement, and conservation of rare species as a means of gaining large numbers of oocytes for in vitro production of embryos. Clinical IVM has the focus of providing an alternative source of developmentally competent oocytes without the administration of significant levels of exogenous follicle stimulating hormone (FSH). These oocytes are then able to undergo routine IVF and embryo transfer (ET) procedures. 

Therefore, IVM is more useful for women whom respond inappropriately to FSH stimulation, particularly women with PCOS who are extremely sensitive to ovarian stimulation and have a high potential for developing of OHSS (4). Also, for non-PCOS cases, IVM could potentially reduce hormonal use associated with IVF, which has both financial and psychological benefits (5). In recent years, many improvements have been made in clinical aspects of IVM treatment which have resulted in better pregnancy outcomes (6). It was shown that pretreatment with FSH in the early days of folliculogenesis phase may increase the number of retrieved immature oocytes and/or maturation potential (7). 

In addition, hCG priming promotes some GV oocytes to reach the maturity and increases the IVM outcomes (8). Despite increased pregnancy rates following IVM in recent years, implantation rates are still lower than conventional IVF cycles.

**Laboratory aspects of IVM**

Culture conditions employed for IVM of mammalian oocytes significantly influence fertilization rates and subsequent embryonic development (9). The current rationale for choosing a specific medium for IVM appears to stem largely from methods developed for the culture of other cell types. More research is necessary to determine the specific metabolites needed and optimal culture conditions required for maturing oocytes. Complex culture media, such as TCM-199, contain many components and are designed for somatic cell culture. The major beneficial components for the oocytes seem already to be present in TCM-199 (10). 

However, there is a paucity of information about the effects of culture medium on maturation and developmental competence of immature oocytes. TCM-199 buffered with bicarbonate or HEPES, supplemented with various sera, gonadotrophins and steroids have been widely used in clinical IVM studies. Different energy substrates and nutrients can greatly influence oocyte meiotic and cytoplasmic maturation (11, 12). Chian and Tan designed a new IVM medium and showed that this is beneficial for nuclear and cytoplasmic maturations of immature human oocytes derived from both stimulated and unstimulated IVF cycles (13).

The core of an assisted reproduction program is oocyte quality, because one of the major problems encountered by IVF clinics is to recognize the maturation state of the oocytes obtained from follicles. The cytoplasm of the oocyte is of key interest in oocyte maturation. The best way to improve embryo development is to harvest good quality oocytes. 

It is known that insufficient cytoplasmic maturation of the oocytes will fail to promote male pronuclear formation, and will increase chromosomal abnormalities after fertilization (14). The quality of oocytes has the greatest influence on results of the monospermic fertilization, early embryo development, and implantation. Therefore, the quality of oocytes can be a determining factor in the fertilization process, embryo culture system, and infertility treatment protocols (15-16). 

Good quality oocytes are exceedingly essential for fertilization, especially for successful IVM and subsequent IVF program, which are two major assisted reproductive technology (ART) programs. Routine evaluation of oocyte morphology by phase-contrast microscopy (PCM) is an important predictive marker of oocyte quality, currently utilized for evaluation of the success of a given ART program (17). 

However, low-resolution morphological assessment is not always a sufficient measure of oocyte fertility potential and developmental competence (18). On the other hand, ultrastructural assessments integrated by other investigational approaches, seems effective in evaluation of oocyte quality. Successful fertilization, development and pregnancy have been achieved with immature human oocytes after IVM in some infertility centers (8). However, little is known about what conditions are suitable for IVM of human oocytes, enabling IVF and embryo development in culture. The present review was therefore aimed to investigate the possible effects of fine quality of human oocytes on the success of clinical IVM program.

**Figure 1:**
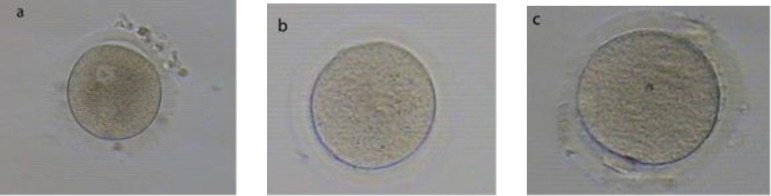
Morphological markers characterizing the meiotic status of human oocytes.

a) Immature germinal vesicle (GV) oocyte.          b) Immature germinal vesicle breakdown (GVBD) oocyte (M1).           .c) Mature oocyte (MII).

**Figure 2:**
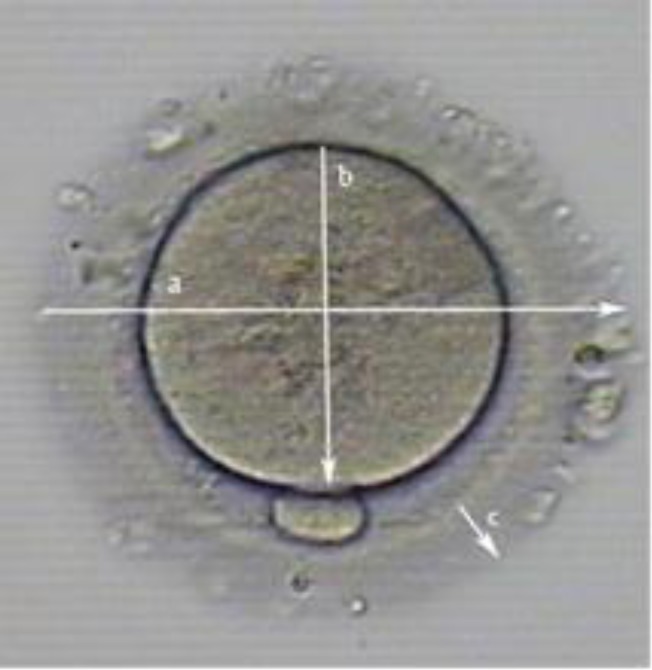
Mormorphometry dimentions of oocytes after IVM

a) Diameter of oocyte           b) Diameter of ooplasm          c) Diameter of ZP

**Figure 3:**
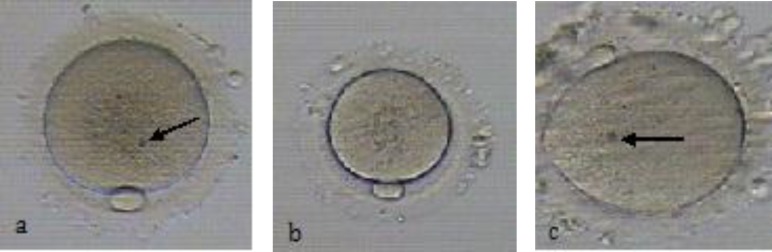
Various morphological abnormalities exhibited by MII oocytes after IVM.

a) Central granularity with refractile body.           b) Wide PVS without debris.           c) Bull’s eye within ooplasm.

**Figure 4:**
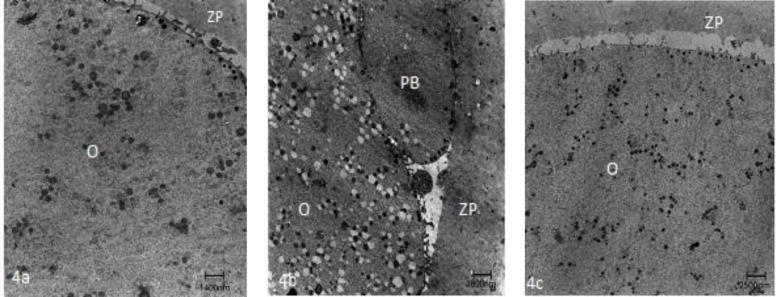
General fine structure of control MII oocyte (A), GV (B) and MI (C) stage oocyte after IVM. O= Oocyte; ZP= Zona pellucida; PB= Polar body

**Figure 5:**
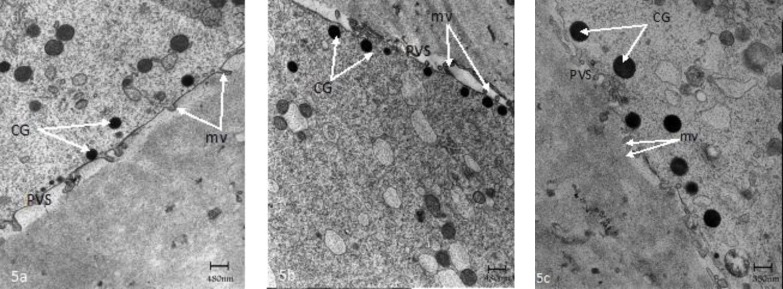
Ultrastructure of control (a) and GV and M-I stage oocyte after IVM (b-c), respectively. mv= microvilli; CG= cortical granules; PVS= perivitelline space.

**Figure 6:**
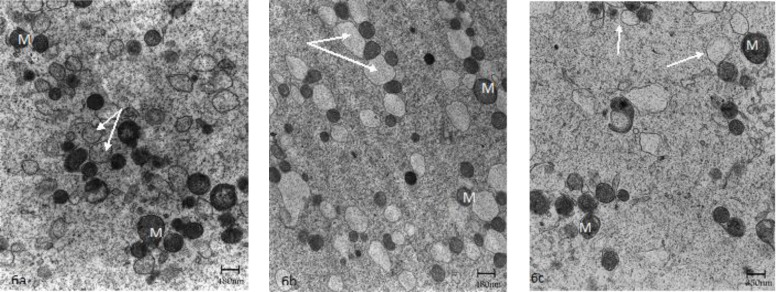
Control oocyte (a), GV and M-I stage oocyte after IVM (b-c), respectively. Mitochondria (M) and vesicles of smooth endoplasmic reticulum complexes (arrows).

**Figure 7:**
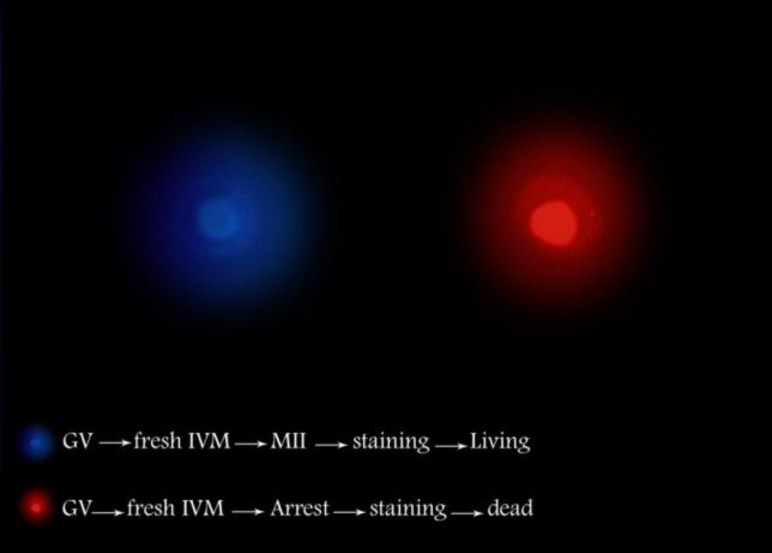
Immunocytochemy of human oocytes. Hoechst propidium iodine (H/PI) nuclear staining for viability assessment.

## Discussion

If oocytes are removed before ovulation from an antral follicle, the separation triggers a pseudo-maturation event leading in general to the completion of the first meiotic division and the second arrest at the metaphase II stage. This was described in 1939 by Pincus (19) and generalized to most domestic mammals by Edwards in 1965 (20). This process called spontaneous maturation is believed to be induced by the removal of an inhibitory factor present in the follicular context (21). The mechanism of such inhibition has been elusive for 40 years, although the impact of cAMP on the inhibitory process has been known during this period (22). 

A recent paper by Zhang *et al* has demonstrated the importance of cGMP in the control of phosphodiesterase-3 involved in the crucial decrease of intra-oocyte cAMP and meiotic resumption (23). This may also explain the reason cumulus-enclosed oocytes cannot generate this inhibitory signal outside of the follicle and the difference between induced and spontaneous maturation. 

Sirard *et al* pointed out the influence of the oocytes quality on the resumption of meiosis, embryo development, implantation, and healthy birth (24). Cytoplasm changes which accompany the oocyte growth, include mRNA transcription and protein synthesis (25). These processes are necessary for the oocyte meiotic maturation, activation of the zygotic genome, and blastocyst formation (26). Oocyte is a complex cell with many organelles, each of which must be in the appropriate state for the maturation of cell (27). 

Any dysfunction or dislocation of the oocyte components, such as meiotic spindle, cortical granules, or mitochondria, can decrease the oocyte viability and has a crucial impact on embryo development (28). The scientists considering the importance of the oocyte quality in the development of embryo have started intensively to search for reliable criteria, which were divided into morphological, cellular and molecular criteria. 

**Oocyte maturation in IVM**

IVM involves removal of oocytes and their cumulus cells from follicles before ovulation occur, after which meiotic maturation begins spontaneously in culture (1). The basal IVM rate of GV oocytes collected from stimulated cycles differ widely between studies. Differing reports of IVM rates are probably due to several factors, including IVM medium, source of oocytes (unstimulated vs stimulated cycles) and whether or not cumulus cells are retained with the oocyte (29). 

Previous studies indicated the IVM rate (~37%) of GV oocytes collected from stimulated cycles (30, 31). However, recent studies reported the IVM rate of about 60% for GV oocytes collected in stimulated cycles (32, 33). Recently, Mohsenzade *et al* reported that there was no significant relation between patient's age and the rates of oocyte maturation (33). They also showed that the maturation rate after IVM was higher in patients with male infertility than in cases with ovarian infertility. Chian and Tan reported the IVM rate of 70.8% for immature M-I oocytes collected from stimulated cycles (13). 

Child *et al* reported that immature oocytes derived from normal ovaries or women with PCO/ PCOS, when using hCG priming, have a similarly high potential for maturation, fertilization, and cleavage. They also recognized that mode of anesthesia used during oocyte retrieval may affect oocyte maturation rates (34). 

Typical pregnancy rates with IVM are now 30-35% per retrieval with 10-15% implantation rates (35). Higher pregnancy rates are seen in women with higher antral follicle counts as well as thicker endometrium. There is no compelling evidence thus far that the risk of congenital malformations is higher with IVM, but as with any new development in ART, further surveillance is necessary. The process of IVM could affect the oocytes quality as any intervention in their growth phase would affect oocyte maturation and subsequent embryo development (36).

**Oocyte quality**

The quality of oocytes definitely impacts early embryonic survival, the establishment and maintenance of pregnancy, and fetal development. Quality or developmental competence is acquired during folliculogenesis as the oocyte grows and during the period of oocyte maturation. ART involving collection of immature oocytes followed by IVM results in oocytes with reduced developmental competence. Although meiosis, or nuclear maturation, may be completed successfully, there are varieties of other processes occurring within the ooplasm that are required for complete developmental competence. Successful completion of these events is independent of nuclear maturation and is collectively referred to as cytoplasmic maturation. 

Cavilla and associates, using computerized micrographic images of human oocytes in culture for IVM, confirmed the findings of Durinzi *et al* which suggested the potential of noninvasive oocyte measurements as predictors of subsequent ability to develop. Cavilla *et al* also evaluated via IVM, 86 oocytes obtained from women with PCOS (37-39). The diameter of the 28 oocytes that survived and progressed in IVM ranged from 103 and 121 µm, with a median of 108 µm. They concluded that oocytes that were larger when collected for IVM had a greater probability to mature. During the growth phase, the diameter of human oocytes increases from 30-110 µm over a period of 8 weeks (40). The diameter of IVM oocytes, excluding the zona pellucida (ZP), usually is 110-120 µm, while the thickness of the ZP is 15-20 µm (41). 

Ubalidi *et al* evaluated morphology of oocytes and reported that alterations, such as fragmented polar body and large PVS are rather higher than other abnormalities, 49% and 32%, respectively (42). Recently, Nazari *et al* evaluated morphology and morphometric of human immature oocytes after IVM. They showed that the rates of fragmented polar body and smooth endoplasmic reticulum (SER) were approximately similar to Ubalidi *et al*. They also reported that other defects, such as Bull’s eye, thick ZP, SER and dark ZP were not observed in human oocytes matured under in vitro condition (Figures 1-3) (32).

An oocyte that has not completed cytoplasmic maturation is of poor quality; thus unable to successfully complete normal developmental processes. However, the cellular mechanisms that impart oocyte developmental competence are entirely unclear. Until the mechanisms involved in oocyte quality are elucidated, any effort to use ART in the treatment of infertility will be inefficient at best (43). Oocytes matured in vitro often have altered energy metabolism and reduced developmental potential. This may reflect a deficiency in the maturation medium, the intrinsic ability of the oocyte itself, or both. However, unless the oocyte is able to correctly control its metabolism, it will exhibit reduced viability. Regulation of nutrient metabolism is controlled at several levels, including substrate availability in the environment, transport systems in the plasma membrane, and enzyme activity and regulation (44). These mechanisms may be critical in the oocyte to create an environment supportive of nuclear and cytoplasmic maturations. 

**Ultrastructure in IVM**

Electron microscopy techniques are the best tools to explore the cell fine structure, but involve important limitations. They rely on expensive technology and highly trained personnel. They can be hardly used for the analysis of large numbers of samples, being the process of specimen preparation and observation lengthy and almost entirely manual. In addition, the samples are fixed for ultra-observation. Regardless, they can provide accurate and extensive information on the fine structure of the cell and its organelles (45). Intracellular damage under culture conditions can be evaluated only by transmission electron microscopy (TEM) (46, 47). 

In one recent study, the human immature oocytes were cultured for 36h and investigated by TEM (48). The ultrastructural examination revealed an ooplasm with characteristics of in vivo matured oocytes without any signs of degeneration of aging vacuolization. The normal features included the presence of dense cortical granules correctly positioned under oolemma and small and large SER vesicles with associated mitochondria (Figure 4). Also, visibles were SER tubular aggregates with associated mitochondria in the cortical region (Figure 5). The main abnormality was related to numerous large microvilli (MV) complexes as compared with matured oocytes by in vivo, that may be due to IVM technique (Figure 6) (48). 

TEM also distinguishes morphological differences between apoptosis and necrosis (49). Morphological characteristics of apoptosis include deletion of single cells, cell shrinkage, membrane blabbing, nuclear condensation, cell disruption into small membrane enclosed fragments (apoptotic bodies), and phagocytosis by neighboring cells. Necrosis is characterized by death of cell groups, loss of membrane integrity, cell swelling, lysosomal leakage, and clumpy ill-defined aggregation of chromatin (49).

**Immunocytochemical methods**

Various immunocytochemical methods for the evaluation of DNA division or GC proliferation have been established. The first identifies the expression of proliferating cell nuclear antigen (PCNA) in GC during culture (50). PCNA is a nuclear protein that plays an essential role in cell cycle regulation and is a mediator of cell proliferation. These characteristics make it a useful marker for proliferating cells. The expression of PCNA in GC of primordial follicles correlates with the initiation of folliculogenesis, and it appears in these cells only when they begin to grow (51). 

Miyara *et al* suggested that changes in chromatin configuration in human oocytes vary with size. They established three oocyte size categories (small, intermediate, and large), with the largest oocyte size being associated with GVBD (52). Combelles *et al* also reported the relevance of oocyte size, which in their study varied between 106µm and 115µm. They also established two oocyte size categories related to oocyte competence (29). It can be said that the larger the oocyte is, the earlier the ovum matures, but small oocytes are associated with atresia and are thought to be incompetent (29, 53). 

Escrich *et al* reported that differences in oocyte size may be due not only to chromatin stage, but also to variations in the ovarian hyperstimulation protocols. They also proposed a predictor method based on four morphologic and morphometric variables (nucleoplasm appearance, nucleolus position, continuity of nuclear envelope, and oocyte size) that provide information about the chromatin condensation stage of the oocyte (54). For assessment of viability of human immature oocytes, Zhang *et al *developed a supravital immunostaining using Hoechst propidium iodide (H/PI) nuclear staining. They found that 44% of their cases were viable following H/PI florescent staining (Figure 7) (55).

The polarization microscopy has entered the realm of clinical ARTs and has become useful in assessments of oocyte quality for human embryo production (56). To gain further insight into the biomechanical properties of spindle micro tubules and chromosomes in human oocytes, high resolution confocal microscopy has been used to define structural properties of human oocyte meiotic spindles related to aneuploidy during advancing maternal age (57).

## Conclusion

Human immature oocytes subjected to the IVM show good general and fine structural preservation. In particular, the absence of cytoplasmic vacuolization (form of cell damage) seems the most relevant marker of quality in IVM program. Taking into account the important clinical significance, researchers need to continue pursuing their studies until morphologically competence oocytes are obtained for use in IVM. Once, success of IVM is highlighted, the availability of this treatment will probably be demanded not only by PCO patients, but also by other infertile cases.
